# Impact of oncologic pathology (OP) in the evolution of severe sepsis (SS) in the critically ill patients (CIP)

**DOI:** 10.1186/2197-425X-3-S1-A247

**Published:** 2015-10-01

**Authors:** J Ruiz Moreno, E González Marín, MJ Esteve Paños, R Corcuera Romero de la Devesa, M Moral Guiteras, M Juliá Amill, N Suárez Álvarez, N Conesa Folch, F Baigorri González, A Artigas Raventós

**Affiliations:** Critical Care Department, QuirónSalud Hospital Universitario Sagrat Cor, Barcelona, Spain; Critical Care Department, Hospital de Clínicas de Sabadell & QuirónSalud Hospital Universitario Sagrat Cor, Sabadell, Spain

## Intr

It is considered that the severity of septic CIPs is higher than the overall CIPs requiring ICU admission. Nevertheless, the impact of the OP over the severity of the SS perhaps has not been sufficiently analyzed and evaluated.

## Objectives

To evaluate the impact of the OP in the evolution of the SS of the CIPs.

## Methods

· Study: prospective, analytical, longitudinal, and observational

· Period: January 1-2011 / June 30-2014 (42 months)
· **SETTING.** Medical/Surgical ICU
· Population: 2559 CIPs admitted consecutively to the ICU; sample: 484 CIPs with SS.

· Exclusión criteria: CIPs < 16 y., major burn CIPs, incomplete clinical documentation, and voluntary discharge.

· Variables analyzed:

a) Age

b) Hospital mortality

c) Case - mix: metabolic acidosis, total parenteral nutrition, intra-abdominal pressure (IAP), blood products, cultures, cardiac output, renal replacement therapy (RRT), advanced life support (ALS), FGC, FBC,

e) Organ dysfunction: SOFA and LODS

f) Limitation of life support (LLS).

· Statistical analysis: Ji squared and contrast of means (Student's t)

· Limitations of the study: absence of critically burned patients and pediatric CIPs

## Results

Global CIPs: 2559; sepsis CIPs: 484; non sepsis CIPs: 2075

SOFA: Global (2.70), septic CIPs (5,32), non-septic CIPs (1,90)

LODS: Global (1.37), septic CIPs (2,78), non-septic CIPs (0,94)

See Tables 1 and 2.

**Table 1:** Table 1

	Global	%	SS	%	SS with OP	%	SS witout OP	%	p value
N	2559	100	484	18,9	130	26,9	354	73,1	
Age	65,88	16,7	73,5	13,1	73,18	11,4	73,64	13,7	NS
Mortality	182	7,1	120	24,8	39	30,0	81	22,9	NS
RRT	91	3,6	70	14,6	20	15,8	50	14,1	NS
TPN	467	18,2	184	38,0	90	69,3	94	26,5	0,0001
IAP	136	5,3	101	20,8	37	28,6	64	18,1	0,012
Metb acid	955	37,3	368	76,0	113	86,9113	255	72,0	0,0006

**Table 2:** Table 2

	Global	%	SS	%	SS with OP	%	SS witout OP	%	p value
Blood products	500	19,5	216	44,6	83	63,8	133	37,6	0,0001 NS NS
Cultures	689	26,9	456	94,2	119	91,5	337	95,2	NS
Pericardiocent.	8	0,3	3	0,6	2	1,5	1	0,9	NS
ALS	85	3,3	42	8,7	16	12,3	26	7,3	0,085
FGC	54	2,1	28	5,8	8	6,1	20	5,6	NS
FBC	61	2,3	47	9,7	12	9,2	35	9,9	NS
LLS	220	8,6	122	25,2	41	31,5	81	22,9	0,0518

## Conclusions

1) The OP conditions not age or mortality of CIPs with SS.

2) Metabolic acidosis and the need of TPN, IAP and blood products are higher in the SS with OP.

3) Cultures, RRT, ALS, FGC, and FBC are applied equally in both groups.

4) The LLS is applied more in the SS with OP.
